# Study on Microstructure and Wear Resistance of Ni60-WC Composite Coatings Fabricated by Plasma–Laser Hybrid Cladding

**DOI:** 10.3390/ma19081572

**Published:** 2026-04-14

**Authors:** Jiacheng Li, Jinyi Wang, Zhaoqing Zhan, Xiaopeng Zhao, Haoli Jiang, Fanlu Min, Jianfeng Zhang

**Affiliations:** 1 College of Materials Science and Engineering, Hohai University, Nanjing 210024, China; jcli0318@163.com (J.L.);; 2 CCCC Tunnel Engineering Co., Ltd., Beijing 100102, China; 3 College of Civil and Transportation Engineering, Hohai University, Nanjing 210098, China

**Keywords:** plasma–laser hybrid cladding, Ni60-40 wt% WC composite coatings, microstructure, microhardness, wear resistance

## Abstract

The efficient fabrication of high-quality Ni60-WC composite coatings with low dilution and defect density remains a challenge for wear-critical tunneling cutters. In this study, a plasma–laser hybrid cladding (PLHC) strategy was developed to fabricate Ni60-40 wt% WC composite coatings, and their microstructures and properties were systematically compared with those produced by plasma cladding (PC) and laser cladding (LC). The PLHC coatings exhibit a low dilution rate of 10.7% and an ultra-low porosity of 0.2%, indicating improved metallurgical integrity. Microstructural analysis reveals that the hybrid energy input effectively suppresses WC dissolution and promotes a refined, uniformly distributed hard-phase network within the Ni-based matrix. As a result, the PLHC coatings achieve a high average microhardness of 1187.83 HV_1.0_ and superior wear resistance, with a wear volume of 24.69 × 10^−3^ mm^3^ under a 200 N load, representing reductions of 53.6% and 20.9% compared with PC and LC coatings, respectively. These results demonstrate the effectiveness of plasma–laser hybrid cladding in tailoring the microstructure–property relationship of WC-reinforced composite coatings.

## 1. Introduction

In modern industrial equipment, over 50% of wear failures are caused by abrasive wear, which is the dominant wear mechanism under extreme conditions for equipment such as tunnel boring tools, mining cutters, and stamping dies [1,2]. The WC-Ni composite coatings, which incorporate WC particles into a ductile Ni-based matrix, are of critical importance in modern industrial applications due to their high hardness, excellent wear resistance, impact resistance, and thermal fatigue resistance, making them suitable for demanding service environments [3,4,5,6]. For example, Zhao et al. [7] prepared Ni/WC metal matrix composite coatings on an Inconel 617 substrate by combining thermal spraying and electron beam remelting, which significantly enhanced the comprehensive performance of the coatings. The average hardness of the 16 mA remelted specimen reached 1081.3 HV, approximately 1.47 times higher than that of the thermally sprayed coating. Moreover, it exhibited the optimal corrosion resistance in 3.5% NaCl solution, with the lowest corrosion current density and the highest corrosion potential. Li et al. [5] fabricated Ni60-WC composite coatings on QT500-7 substrates, demonstrating a significant improvement in hardness, corrosion resistance, and tribological properties, thereby prolonging the service life of brake disks.

Although Ni-WC composite coatings possess essentially excellent properties including high hardness, wear resistance, impact resistance, and thermal fatigue resistance, the fabrication of Ni-WC composite coatings is still limited by conventional processing routes. For example, wear-resistant Ni-WC composite coatings have been used for shield machine cutterheads through built-up welding, plasma cladding (PC), and laser cladding (LC). Built-up welding yields coatings with a coarse microstructure and compositional heterogeneity. In addition, extended heating induces WC decarburization, forming brittle phases that compromise the hardness and wear resistance of the coating [8,9].

On the other hand, although PC achieves higher deposition rates, it imposes greater thermal loads on substrates and causes higher dilution, which can degrade coating performance. The LC process produces dense coatings but requires tightly controlled powder characteristics and offers limited deposition rates, increasing processing costs. Based on this consideration, many researchers have tried to conquer this dilemma by trying different process methods. For example, Sun et al. [10] produced a Cu_11.85_Al_3.2_Mn_0.1_Ti coating by applying laser remelting to plasma-clad layers, and Lan et al. [11] produced an FeCoCrNiAl_0.5_ coating through plasma nitriding followed by laser cladding. It is clear that LC remelting can refine the grain structure of PC coatings and reduce the porosity of LC coatings to some extent, but the coatings still exhibit issues such as the sedimentation and agglomeration of hard particles. Furthermore, repeated thermal cycles have been found to increase the dilution rate, resulting in degraded wear resistance. To address the limitations of the above-mentioned processes, we aim to overcome the drawbacks of single-process cladding by employing dual-heat-source synchronous cladding to obtain WC-reinforced composite coatings with excellent microstructural uniformity and wear resistance. A laser is introduced as a secondary energy source to precisely control the molten pool and suppress WC sedimentation, while reducing substrate overmelting and dilution rate under the premise of maintaining equivalent heat inputs.

In this study, in comparison with PC and LC, we introduced a new plasma–laser hybrid cladding (PLHC) strategy that employed a laser as a secondary energy source to augment conventional plasma deposition. Unlike previously reported hybrid or remelting-assisted techniques, our PLHC employs simultaneous plasma and laser irradiation in a single pass. This allows the plasma beam to provide high thermal input for efficient deposition and hard-phase precipitation, while the laser beam precisely stabilizes the molten pool, suppresses WC sedimentation, and refines the microstructure without an extra post-treatment step. The Ni60-40 wt% WC composite coatings were fabricated on steel substrates by PLHC, and its dilution rate, porosity, microhardness, and microstructure distribution were systematically investigated. Furthermore, the wear resistance was characterized, and the underlying enhancement mechanism was elucidated.

## 2. Materials and Methods

### 2.1. Coating Preparation

The Ni60-40 wt% WC composite coatings were fabricated using Ni60 alloy powder (99.8% purity, Quanyue Metal Materials, Mianyang, China) as the matrix and spherical cast tungsten carbide (WC 99.8% purity, Quanyue Metal Materials, Mianyang, China) as the ceramic reinforcement. The composite powder was prepared by mixing the constituents in a three-dimensional mechanical mixer (HSYH-5, Xinfei Dry Equipment Factory, Nanjing, China) for 24 h with 40 wt% spherical WC, followed by drying at 100 °C for 1 h. Before processing, the steel substrate was cleaned with a laser marking system (XC-XFC 20, Xingcheng Laser Technology, Qingdao, China) to remove surface oxides and impurities that could compromise coating adhesion. Subsequently, the substrate was preheated to 200 °C using a laser power of 1200 W to mitigate thermal stress and suppress crack formation.

**Figure 1:**
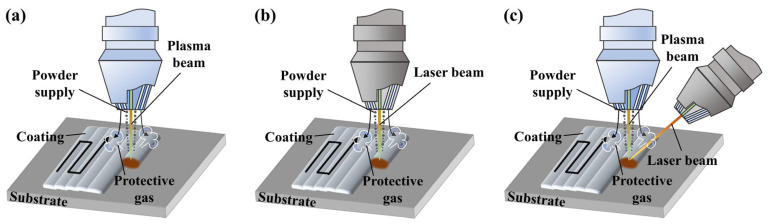
Schematic diagram of different processes for fabrication of Ni60-40 wt% WC composite coatings. (**a**) PC, (**b**) LC, (**c**) PLHC.

The self-assembled dual-heat-source novel plasma–laser hybrid cladding process employs a plasma beam as the primary heat source. The plasma cladding head (TETRIX 552 AC/DC, IDA New Technology Power Supply, Kunshan, China) emits powder around its periphery while the plasma beam strikes its center. Simultaneously, the laser (P6000, Guanghui Laser Technology, Shanghai, China) emits a laser beam at a 45° angle as the secondary heat source, achieving dual-axis composite cladding. The schematic representation of the innovative dual-heat-source plasma–laser composite cladding process is demonstrated in Figure 1. The deposition rate (g/min) is obtained by multiplying the powder feed rate by the powder utilization efficiency. The powder utilization efficiency is calculated as the net mass gain of the deposited material divided by the mass of the powder before cladding. The net mass gain is precisely measured by the gravimetric method as the difference in the substrate mass before and after the laser cladding experiment.

After the coating treatment, the as-deposited samples were cut to 10 mm × 10 mm × 12 mm using wire electrical discharge machining. Sectioned samples were ultrasonically cleaned in alcohol for 30 min, then mounted using phenolic resin at 130 °C under a pressure of 30 MPa and ground with silicon carbide metallographic abrasive paper. Subsequent polishing was performed using a metallographic polisher, with progress monitored via optical microscopy until a scratch-free mirror finish was attained. Finally, the specimens were ultrasonically cleaned in alcohol and dried in cool air.

To evaluate the differences in forming quality and performance among Ni60-40 wt% WC composite coatings fabricated by PC, LC, and PLHC, and highlight the benefits of the PLHC process, equivalent heat inputs of 300 J/mm were maintained, as shown in Table 1. For this purpose, *Q_Total_*, *Q_laser_* and *Q_arc_* were calculated through the following Equations (1)–(3) [12]:

(1) $$Q_{Total}=Q_{laser}+Q_{arc}$$

where $Q_{Total}$ was the total heat input (J/mm) for the different processes, $Q_{laser}$ was the laser heat input (J/mm), and $Q_{arc}$ was the plasma heat input.

(2) $$Q_{laser}=\frac{P_{laser}}{v}$$

(3) $$Q_{arc}=\frac{U_{arc}\times I_{arc}}{v}$$

where $P_{laser}$ was the laser average power (W), $U_{arc}$ was the plasma voltage (V), $I_{arc}$ was the plasma current (A), and v was the scanning speed (mm/s).

### 2.2. Microstructural Characterization

Cross-sectional images were obtained using an optical microscope (BX51M, Olympus, Tokyo, Japan) and subsequently analyzed with ImageJ software (ImageJ 1.54g Wayne Rasband and contributors National Institutes of Health, Bethesda, MD, USA). Coating porosity, defined as the ratio of pore volume to the total volume of the coating, was quantitatively evaluated using a standardized metallographic method. For each coating sample, fifteen randomly selected views were systematically examined to ensure representative sampling for the average. Furthermore, the dilution characteristics of single-track clad layers were investigated using a field-emission scanning electron microscope (Quanta250FEG, Fei, Hillsboro, OR, USA), and calculated according to Equation (4) [13] which is as follows:

(4) $$\eta =\frac{h}{h+H}\times 100\%$$

where *η* was the dilution rate (%), *h* was the cladding depth minus the cladding height, and *H* was the cladding height.

The phase composition of Ni60-40 wt% WC composite coatings were characterized by X-ray diffraction (D8-Advance, Bruker, Ettlingen, Germany) using Cu Kα radiation at 40 kV and 40 mA, with scans collected from 30° to 100° (2θ) at a rate of 4°/min. Microstructural examination was conducted using a scanning electron microscope (Nova Nano SEM 450, Fei, Hillsboro, OR, USA), with chemical composition determined by energy-dispersive X-ray spectroscopy (Oxford X-act, Oxford Instruments, Abingdon, UK).

### 2.3. Properties Testing

Microhardness of cross-sectional Ni60-40 wt% WC composite coatings was measured using a Vickers hardness tester (HXD-1000TM/LCD, Taiming Optical Instrument, Shanghai, China) under a 1000 g load with 15 s dwell time. An indentation grid was applied with points spaced 200 μm apart horizontally (5 columns total) and 100 μm apart vertically from the top surface of Ni60-40 wt% WC composite coatings into the substrate. The five measurements on each horizontal level were averaged to represent the hardness at that depth.

Tunnel boring machine (TBM) cutter rings and mining tools typically operate under dry sliding conditions at room temperature without external lubrication. The primary wear mechanisms during service are abrasive wear caused by hard rock particles and adhesive wear under high loads. When a TBM operates in complex environments containing hard rock, the local compressive stress on the cutter ring surface typically ranges from 2.0 to 3.0 GPa. The contact stress is calculated according to the following Hertzian contact stress formula:

(5) $$P_{\max}={\left(\frac{6F{E^{*}}^{2}}{\pi^{3}R^{2}}\right)}^{\frac{1}{3}}$$

where *F* is the load (N), $E^{*}$ is the equivalent elastic modulus (GPa), and *R* is the spherical radius (m). When the load force is set to 200 N, the contact stress between the friction pair and the coating is approximately 2.9 GPa. Therefore, this study selects 200 N as the maximum load for friction and wear testing. In this study, the wear resistance of the composite coatings was evaluated using a multi-functional tribological tester (MFT-3000, Rtec, San Jose, CA, USA) to simulate actual working conditions. Wear tests were performed using a reciprocating sliding mode, with a Si_3_N_4_ ceramic ball (12.7 mm diameter) as the friction pair. The specific test parameters included a wear stroke of 2 mm, oscillation frequency of 5 Hz, test duration of 30 min, average sliding speed of 20 mm/s, and total sliding distance of 36 m. The wear resistance of the samples was assessed under three high load conditions, 100 N, 150 N, and 200 N, with each test repeated three times to ensure data reliability. The three-dimensional surface profiler (UP-3000, ITECH Instruments, Ewing, NJ, USA) was used to measure the two-dimensional profiles, three-dimensional morphologies, and wear volumes of the wear scars. The obtained three-dimensional morphologies were processed using the accompanying software, and the wear rate was calculated based on the wear volume, sliding distance, and load [14].

## 3. Results and Discussion

### 3.1. Phase Composition and Morphology

#### 3.1.1. Morphology of the Raw Powders

**Figure 2:**
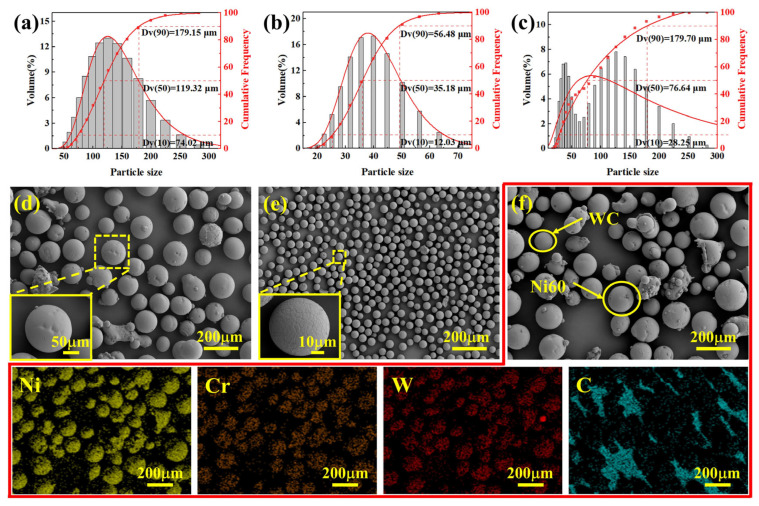
Particle size distribution, morphology and element mapping of raw powders. (**a**,**d**) Ni60, (**b**,**e**) WC, (**c**,**f**) Ni60-40 wt% WC.

Figure 2a–c show the particle size distributions of the Ni60, WC, and Ni60-40 wt% WC powders, respectively. Figure 2d–f present the SEM images of the Ni60, WC, and Ni60-40 wt% WC powders, along with the EDS spectrum of the Ni60-40 wt% WC powder. Both powders display high sphericity and are largely free of voids or defects. The presence of a limited number of satellite particles in the Ni60 powder confirms its good flowability. The Ni60 alloy and WC powders have median diameters of 119.32 μm and 56.48 μm, respectively, while the resultant Ni60-40 wt% WC mixed powder has a median diameter of 76.64 μm.

#### 3.1.2. Phase Composition of the Coatings

**Figure 3:**
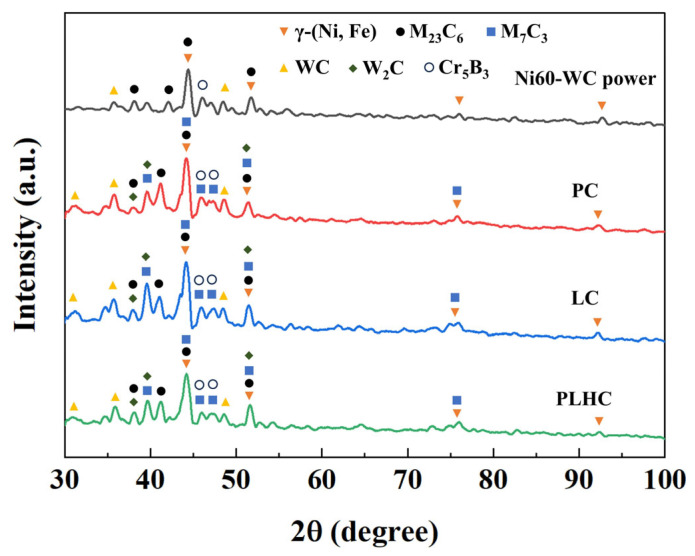
XRD patterns of the Ni60-40 wt% WC powder and the Ni60-40 wt% WC composite coatings fabricated by PC, LC, and PLHC.

Figure 3 shows the XRD patterns of the Ni60-40 wt% WC powder and the corresponding Ni60-40 wt% WC composite coatings fabricated by PC, LC, and PLHC. The phase composition of the Ni60-40 wt% WC composite coatings remains nearly identical across the different cladding processes, with differences observed only in the peak intensities and positions. The Ni60-40 wt% WC composite coatings comprise γ-(Fe, Ni), WC, W_2_C, M_23_C_6_, and M_7_C_3_ phases [15,16]. The γ-Fe phase originates from interdiffusion and elemental exchange between the steel substrate and coating during high-temperature processing. Under high energy density, WC particles fragment and decompose, releasing W and C atoms which subsequently form W_2_C [17]. Owing to the stronger affinity of Cr for C than W, Cr preferentially binds with C in the molten pool, yielding hard phases such as Cr_23_C_6_ and Cr_7_C_3_ [15]. These chromium carbides demonstrate superior thermal stability compared to WC, particularly in oxidative environments, exhibiting reduced tendencies toward decomposition.

#### 3.1.3. Dilution Rate and Porosity

**Figure 4:**
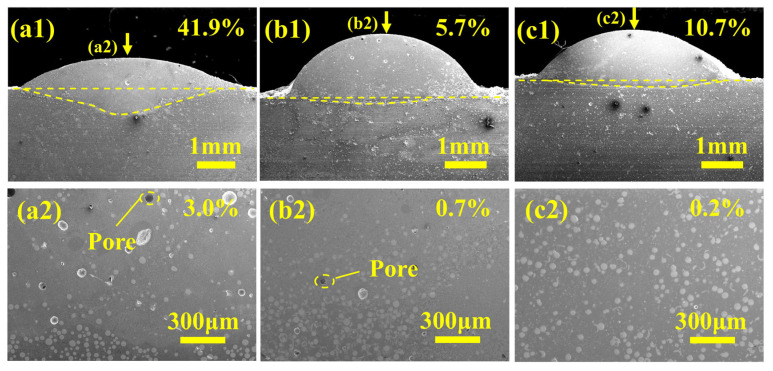
Cross-sectional and top-surface morphology of the Ni60-40 wt% WC composite coatings fabricated by different processes. (**a1**,**a2**) PC, (**b1**,**b2**) LC, (**c1**,**c2**) PLHC.

Figure 4(a1–c1) present the dilution rates of Ni60-40 wt% WC composite coatings fabricated by PC, LC, and PLHC. The measured dilution rates for PC, LC, and PLHC Ni60-40 wt% WC composite coatings were determined to be 41.9%, 5.7%, and 10.7%, respectively. Excessive iron dilution due to a high dilution rate in PC reduces hardness and wear resistance, but ensures strong metallurgical bonding. LC has a low dilution rate, retaining high-hardness components while increasing the risk of coating spalling. PLHC achieves an optimal dilution rate, fully realizing strong bonding with the substrate without compromising coating performance. The elevated dilution rates in PC Ni60-40 wt% WC composite coatings stem from their lower energy density and broader heat-affected zone, which promote deeper heat penetration into the substrate and consequently enhance substrate melting. By contrast, the LC Ni60-40 wt% WC composite coatings exhibit markedly lower dilution, resulting from their high energy density and rapid thermal cycles that concentrate energy at the surface and limit subsurface thermal penetration. The PLHC Ni60-40 wt% WC composite coating achieves an optimal dilution rate, superior bonding strength and a denser microstructure compared with single-process variants [18,19,20]. The plasma beam rapidly deposits a base layer through high heat input, enhancing coating adhesion, while the laser beam combines low heat input with tight focus to precisely treat the substrate surface, limiting intermixing between materials and constraining the heat-affected zone [21]. The combination of high-melting-point WC with low-melting-point Ni60 powders in Ni60-40 wt% WC composite coatings enables uniform deposition under dual-heat-source action, avoiding WC sedimentation and excessive melting of the steel substrate into the upper composite coating [20,22,23].

Figure 4(a2–c2) present the porosity distribution in Ni60-40 wt% WC composite coatings fabricated by PC, LC, and PLHC. The measured porosity values are 3.0% for PC, 0.7% for LC, and 0.2% for PLHC. Porosity originates from gas entrapment or solidification shrinkage during rapid cooling [24,25]. PC exhibits relatively high porosity due to insufficient powder melting, whereas LC achieves lower porosity through rapid melting, metallurgical bonding and precise energy control. Compared with single-process cladding, PLHC enables multidimensional pore suppression by combining the slow solidification of plasma cladding, which improves encapsulation of refractory carbides within the Ni60-based matrix to prevent micro-pores from unmelted materials, with the precise energy control of laser cladding that refines grains and minimizes solidification shrinkage pores [26].

#### 3.1.4. Microstructural Morphology Analysis

**Figure 5:**
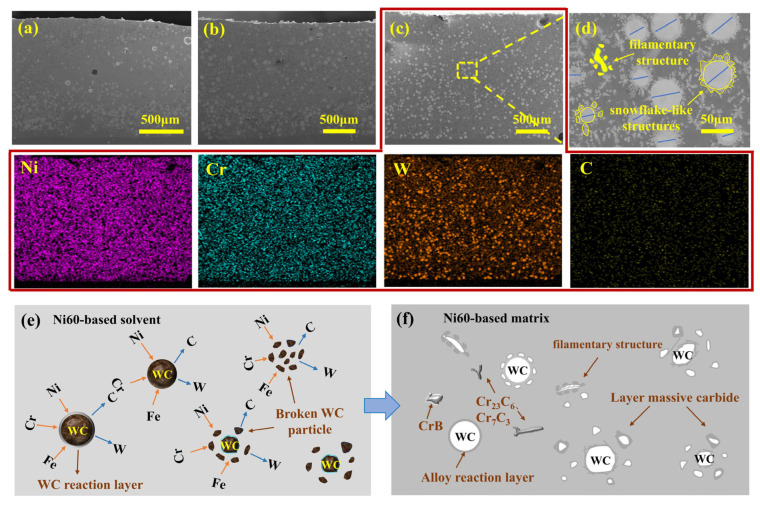
WC distribution and dissolution-diffusion mechanism in Ni60-40 wt% WC composite coatings. (**a**) PC, (**b**) LC, (**c**–**f**) PLHC.

Figure 5a–d present the surface morphology of PC, LC, and PLHC Ni60-40 wt% WC composite coatings, together with the elemental mapping of PLHC Ni60-40 wt% WC composite coatings. The deposition rate of the PC composite coating is approximately 4.18 g/min, with a thickness of about 1.24 mm; the LC composite coating exhibits a deposition rate of approximately 5.08 g/min and a thickness of about 1.72 mm; and the PLHC composite coating shows a deposition rate of approximately 5.58 g/min, with a thickness of about 1.91 mm. Cross-sectional analysis demonstrates that the PLHC Ni60-40 wt% WC composite coating exhibits the most uniform WC distribution, in contrast to the pronounced agglomeration and stratification observed in the single-process Ni60-40 wt% WC composite coatings. Figure 5c and its corresponding EDS spectrum demonstrate a coherent distribution of Ni, Cr, W, and C elements that aligns with the observed microstructure. The uniform dispersion of W and C attests to homogeneous WC diffusion throughout the Ni60-40 wt% WC composite coatings, despite the characteristically faint C signal arising from the low detection sensitivity typical of light elements. Figure 5d quantitatively analyzes the WC size distribution in the PLHC coating. The dissolved WC particles have a median diameter of 45.09 μm, which is significantly smaller than that of the original powder, indicating that the diffusion and dissolution of WC contribute to microstructural refinement. The dual-energy input promotes controlled partial dissolution of WC particles, leading to the formation of a continuous filamentary carbide network that acts as a load-bearing skeleton within the Ni60 matrix. This microstructural architecture is fundamentally different from the simple agglomeration or sedimentation observed in PC and LC coatings. Carbon liberated from fragmented WC particles reacts with metallic species to form new carbides, in agreement with the microstructural observations. While partially dissolved WC particles retain snowflake-like architectures with core–shell features, released fragments develop filamentary networks that collectively enhance the wear resistance of Ni60-40 wt% WC composite coatings. Under the applied load, the hard WC particles and carbide network bear most of the compressive stress, preventing direct contact between the softer Ni60 matrix and the interface. This explains the higher hardness and more stable hardness distribution of the PLHC composite coating compared to PC and LC. The Ni60-40 wt% WC composite coatings’ evolution mechanism during the PLHC process (Figure 5e–f) illustrates the sequential dissolution-diffusion and fracture-dissolution processes of WC particles. In the molten pool, WC particles undergo erosion by the Ni60-based solvent, leading to the interdiffusion of Ni and Cr atoms into the WC particles and the release of W and C into the melt, thereby forming an alloyed layer at the WC interface. Owing to the higher affinity of Cr for C than for W, the liberated C atoms preferentially react with Cr to form Cr_23_C_6_ and Cr_7_C_3_ [15]. Concurrently, mechanical impact from powder collisions and thermal stress promote the fragmentation of some WC particles into finer domains. These fragments experience accelerated dissolution-diffusion with the Ni60-based matrix due to their increased surface area, resulting in the development of a massive carbide layer.

### 3.2. Microhardness

**Figure 6:**
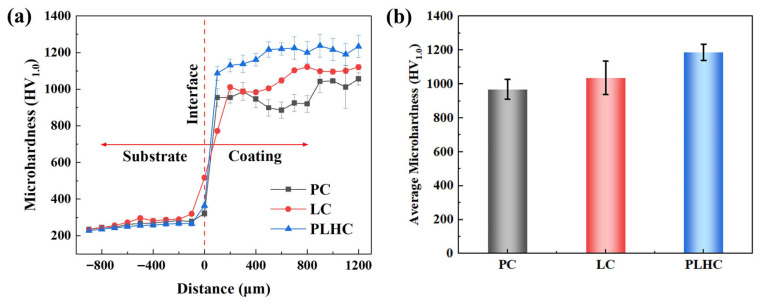
Microhardness of Ni60-40 wt% WC composite coatings. (**a**) Microhardness distribution of cross-sections of Ni60-40 wt% WC composite coatings fabricated by different processes, (**b**) Average microhardness.

Microhardness profiles across the coating cross-sections reveal the influence of cladding techniques on the mechanical properties of Ni60-40 wt% WC composite coatings (Figure 6a). The PLHC Ni60-40 wt% WC composite coating demonstrates the most stable hardness distribution with minimal fluctuations, reflecting its homogeneous WC dispersion. In contrast, the PC Ni60-40 wt% WC composite coating exhibits elevated hardness near the coating–substrate interface, where high-melting-point WC particles settle owing to the limited energy density of the process, while the lower-melting-point Ni60 matrix forms the upper regions. Average microhardness values for three Ni60-40 wt% WC composite coatings are compared in Figure 6b. The PC, LC, and PLHC Ni60-40 wt% WC composite coatings exhibit average microhardness values of 968.79 HV_1.0_, 1037.04 HV_1.0_, and 1187.83 HV_1.0_, respectively, with the PLHC Ni60-40 wt% WC composite coating achieving superior hardness through the synergistic integration of dual heat sources.

The plasma beam provides high thermal input that promotes hard-phase precipitation including W_2_C from WC dissolution and Cr_23_C_6_ from reactions between dissolved C and Cr, while the laser beam imparts microstructural precision. The rapid cooling characteristics of laser processing preserve these hard phases, suppress grain growth and refine their distribution. This combined action of uniform hard-phase dispersion, microstructural refinement, defect minimization and interfacial reinforcement enhances Ni60-40 wt% WC composite coating hardness.

### 3.3. Tribological Properties

#### 3.3.1. Friction Coefficient

**Figure 7:**
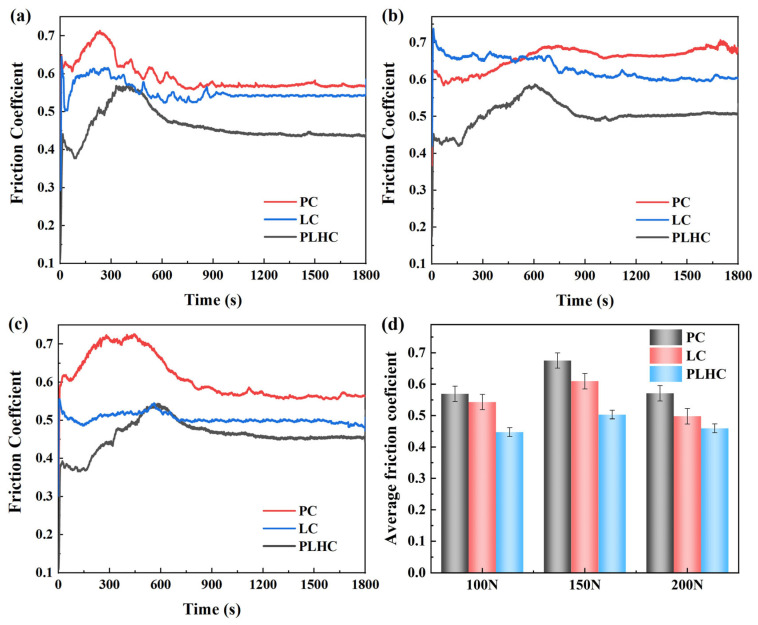
Friction coefficient under different loads of Ni60-40 wt% WC composite coatings. (**a**) 100 N, (**b**) 150 N, (**c**) 200 N, (**d**) Average friction coefficient.

Figure 7a–c present the friction coefficient curves of Ni60-40 wt% WC composite coatings fabricated by PC, LC, and PLHC under different loads. All three Ni60-40 wt% WC composite coatings complete the run-in stage within a short period, during which the friction coefficient rises rapidly to a peak. In the initial wear phase, the limited contact area between the friction pair and coating results in high mean compressive stress, driving the sharp increase in the friction coefficient during the run-in [27,28]. However, after run-in completion, the friction coefficient undergoes significant fluctuations—initially decreasing, then increasing, before decreasing again. In the first phase, rising contact temperature softens the surface layer, increasing the real contact area and distributing shear stress more evenly, thereby reducing the friction coefficient. In the intermediate stage, a hard oxide film forms on the Ni60-40 wt% WC composite coatings’ surface at elevated temperatures, while detached WC particles become embedded in the contact zone, inducing a plowing effect that raises the friction coefficient [7]. In the final phase, the surface oxide layer becomes denser under extreme temperature and wear debris forms a new lubricating medium under high temperature and pressure, improving surface smoothness and again reducing the friction coefficient. During the stable sliding stage, fluctuations in the friction coefficient of Ni60-40 wt% WC composite coatings likely result from strong frictional interactions between dislodged WC particles and the counterpart ball.

While a lower friction coefficient generally correlates with better wear resistance, it serves as an important reference for analyzing tribological behavior rather than a definitive criterion for evaluating wear performance. As shown in Figure 7d, the average friction coefficients of PC Ni60-40 wt% WC composite coatings under 100 N, 150 N, and 200 N loads are 0.57, 0.68, and 0.50, respectively; LC Ni60-40 wt% WC composite coatings show values of 0.54, 0.61, and 0.50; and PLHC Ni60-40 wt% WC composite coatings exhibit 0.44, 0.50, and 0.40. The friction coefficient was calculated in real time by the multi-functional tribological tester based on the ratio of friction force to normal load. To obtain the reported average friction coefficient values, we selected the friction coefficient in the stable region (900–1800 s) for averaging. Under each load condition, PLHC Ni60-40 wt% WC composite coatings consistently demonstrate the lowest friction coefficient. Comparative analysis reveals that the friction coefficients of all three Ni60-40 wt% WC composite coatings initially increase then decrease with increasing load. At higher loads, the lubricating bonding course and oxide scale between the friction pair and coating become compressed and locally fractured, leading to an increase in the friction coefficient. Under 200 N loading, the surface contact transitions from elastic to elastoplastic deformation, rapidly expanding the actual contact area and reducing the load per unit area, thereby lowering the friction coefficient [4,29].

#### 3.3.2. Wear Scar Profile and Volume Loss Analysis

**Figure 8:**
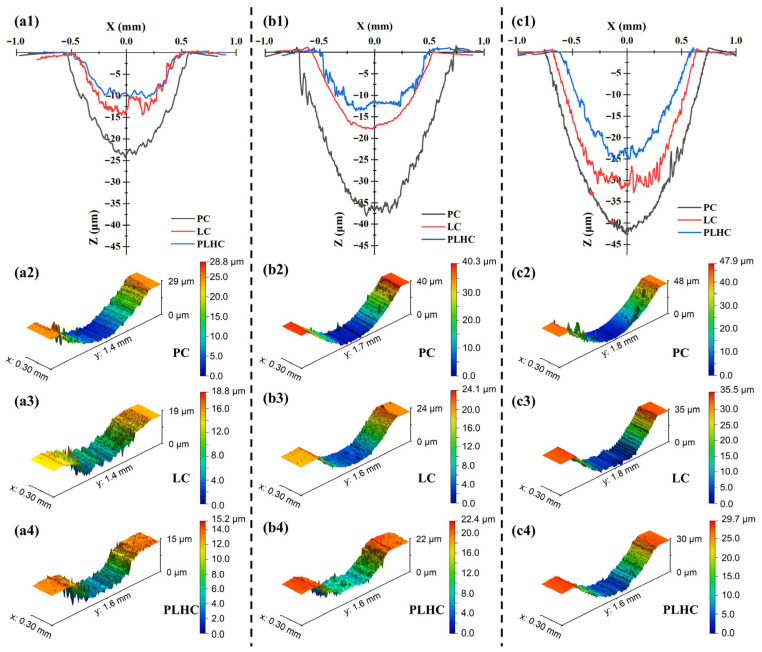
Wear tracks and 3D morphologies of Ni60-40 wt% WC composite coatings under different loads. (**a1**–**a4**) 100 N; (**b1**–**b4**) 150 N; (**c1**–**c4**) 200 N.

Figure 8 displays wear tracks and 3D morphologies of Ni60-40 wt% WC composite coatings fabricated by three processes under different loads. Both wear track depth and width increased with applied load. PC and LC Ni60-40 wt% WC composite coatings showed larger fluctuations in wear track topography than PLHC Ni60-40 wt% WC composite coatings, likely due to inhomogeneous WC distribution that exacerbated plowing. Significant pile-up (above substrate level) occurred around the wear track in all Ni60-40 wt% WC composite coatings, resulting from plastic deformation and debris accumulation. The PLHC Ni60-40 wt% WC composite coatings consistently exhibited the smallest wear track depths across all loading conditions. At the maximum load of 200 N, average wear track depths reached 16.70 μm for PC, 37.99 μm for LC, and 28.38 μm for PLHC Ni60-40 wt% WC composite coatings. With the shallowest wear track (16.70 μm), PLHC Ni60-40 wt% WC composite coatings also demonstrated minimal ball-embedding depth and the narrowest wear track width. These results confirm that the hybrid process produces Ni60-40 wt% WC composite coatings with significantly smaller wear track dimensions under high loads than single-process methods, reflecting its superior wear resistance.

**Figure 9:**
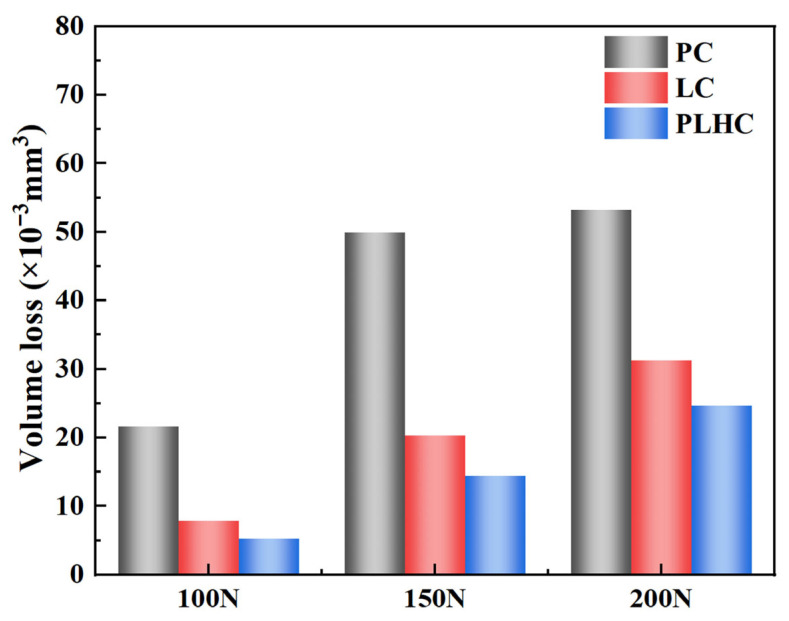
Wear volume loss of Ni60-40 wt% WC composite coatings under different loads.

Figure 9 compares the wear volume loss of Ni60-40 wt% WC composite coatings fabricated by three different processes across different loads. Volume loss increased with applied load for all Ni60-40 wt% WC composite coatings. Under loads of 100 N, 150 N, and 200 N, wear volumes measured 21.65 × 10^−3^ mm^3^, 49.93 × 10^−3^ mm^3^, and 53.26 × 10^−3^ mm^3^ for PC; 7.87 × 10^−3^ mm^3^, 20.34 × 10^−3^ mm^3^, and 31.24 × 10^−3^ mm^3^ for LC; and 5.22 × 10^−3^ mm^3^, 14.44 × 10^−3^ mm^3^, and 24.69 × 10^−3^ mm^3^ for PLHC. The Ni60-40 wt% WC composite coatings fabricated by PLHC showed less volume loss than those from single processes as the ultra-low porosity (0.2%) minimizes this stress concentration, allowing the coating to maintain structural integrity under high loads. This contributes to reduced wear depth and a smoother wear track morphology. The denser microstructure impedes crack initiation and propagation, and its more uniform distribution of reinforcing phases mitigates stress concentration from particle agglomeration and delays brittle spallation. The dual-heat-source setup enables better control of heat input, reduces dilution from the steel substrate, and retains more high-hardness alloy elements. The slower cooling during PC promotes precipitation of hard phases, while rapid laser quenching stabilizes carbide formation, resulting in a wear-resistant “soft matrix + hard particles” structure [30,31] that minimizes material loss, as shown in Figure 5d. In summary, the LC technique produces Ni60-40 wt% WC composite coatings with the least volume loss and superior wear performance.

#### 3.3.3. Wear Morphology and Mechanism

**Figure 10:**
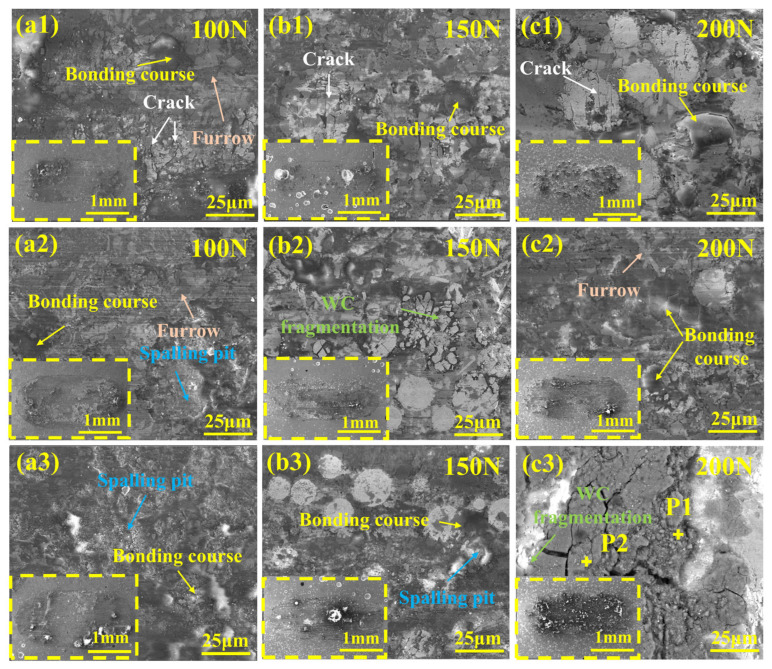
Wear morphology of Ni60-40 wt% WC composite coatings by different processes. (**a1**–**a3**) PC; (**b1**–**b3**) LC; (**c1**–**c3**) PLHC.

Figure 10 presents SEM wear morphologies of Ni60-40 wt% WC composite coatings fabricated by PC, LC, and PLHC under different loads, with corresponding macroscopic tracks shown in the lower-left insets. Surface analysis reveals uniform WC distribution in PLHC Ni60-40 wt% WC composite coatings, in contrast to the surface of PC Ni60-40 wt% WC composite coatings that indicate sedimentation, and the porous, cracked morphology with WC agglomeration in LC Ni60-40 wt% WC composite coatings. Wear morphologies of Ni60-40 wt% WC composite coatings show plowing grooves, oxidized bonding courses and delamination features that intensify with load, confirming abrasive, oxidative and adhesive wear as the dominant mechanisms [32,33]. The O element content at P1 and P2 in Figure 10(c3) is relatively high (as shown in Table 2), and the bonding course forms through the rapid oxidation of wear debris under thermomechanical action between the friction pair and coating, which is characteristic of adhesive wear [34]. The bonding course provides lubrication, reducing both wear rate and volumetric loss. Spalling pits with sharp edges reflect typical brittle fractures, while the laminated and cracked bonding course forms under frictional heating and complex stress. Under 200 N loading, substantial debris accumulation accompanies enhanced WC fragmentation, as shown in Figure 10(a3–c3).

**Figure 11:**
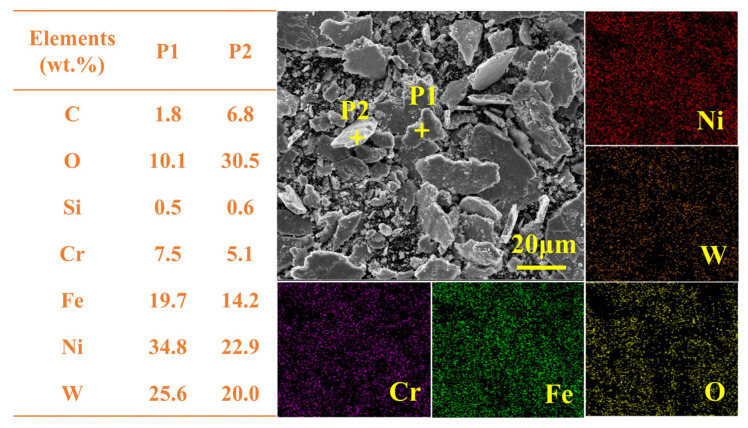
Morphology and element distribution of PLHC Ni60-40 wt% WC composite coating debris.

The accumulated wear debris in Figure 10(c3) was characterized by SEM and EDS, as presented in Figure 11. P1 shows flake-like debris rich in Ni, characteristic of the bonding course generated by adhesive wear. P2, comprising fine white powder with a composition similar to P1 but elevated oxygen, likely represents a fragmented bonding course that underwent oxidative wear before detachment. These findings, together with evidence in Figure 10, establish abrasive, oxidative and adhesive wear as the dominant mechanisms in Ni60-40 wt% WC composite coatings.

**Figure 12:**
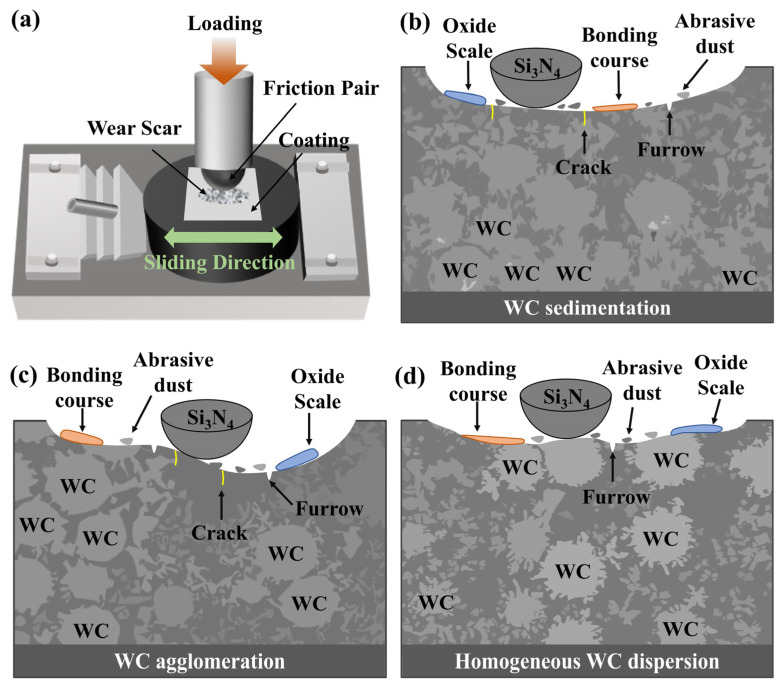
Schematic diagram of wear mechanism of Ni60-40 wt% WC composite coatings. (**a**) Macroscopic process of the reciprocating motion, (**b**) PC, (**c**) LC, (**d**) PLHC.

Figure 12a shows the reciprocating process, in which the local pressure at the friction pair–coating interface exceeds the yield strength of the material, causing plastic deformation [35]. Under the load process, the softer Ni60-based matrix wears preferentially, allowing WC particles to protrude and shield the underlying material. The non-uniform WC distribution in PC and LC Ni60-40 wt% WC composite coatings produces distinct failure modes, where PC Ni60-40 wt% WC composite coatings display severe WC sedimentation with depleted upper-layer particles that result in poor wear resistance and deep tracks, while LC Ni60-40 wt% WC composite coatings develop cracks and fractures in WC agglomerates under stress, exhibiting non-uniform wear resistance across the coating surface. The liberated fragments act as abrasives between the friction pair and coating, intensifying abrasive wear and producing extensive furrows, as shown in Figure 12b,c. By contrast, the uniform WC distribution in PLHC Ni60-40 wt% WC composite coatings enables more homogeneous stress transfer and improved wear resistance [36], as shown in Figure 12d. The uniform filamentary WC network ensures homogeneous stress distribution, preventing localized failure. During wear, Ni60-based debris adheres and mixes with oxides to form a stable surface film and bonding course that lubricates the ball–coating interface and reduces the friction coefficient.

## 4. Conclusions

This work demonstrates the feasibility of a new PLHC process for the fabrication of Ni60-40 wt% WC composite coatings, with a superior morphology compared to single-process variants and a low porosity (0.2%) and appropriate dilution rate (10.7%). It resulted from the dual-heat-source action that balanced clad-substrate stress distribution, regulated dilution, and reduced gas supersaturation at solidification. The PLHC Ni60-40 wt% WC composite coatings demonstrated a uniform hardness distribution, and the highest average microhardness of 1187.83 HV_1.0_ compared to the LC and PC processes. This gain originated from the hybrid process integrating dual-heat-source benefits, which yielded uniform WC dispersion, refined carbide grains, and strong bonding between the Ni60 matrix and WC particles. In addition, the PLHC Ni60-40 wt% WC composite coatings demonstrate superior wear resistance. At the maximum load of 200 N, it shows a wear depth of 24.28 μm and a volume loss of 24.69 × 10^−3^ mm^3^. The dominant wear mechanisms were ascribed to decreased adhesive fatigue and reduced abrasive wear modes due to the refined and uniformly distributed WC grains. This translates into a significantly prolonged service life for tunnel boring cutter rings and mining tools operating under high-load, abrasive conditions.

## Figures and Tables

**Table 1:** Cladding process parameters of Ni60-40 wt% WC composite coatings.

Sample	Plasma Current (A)	Laser Power (W)	Scanning Speed (mm/s)	$Q_{Total}$ (J/mm)	Overlap Ratio (%)	Powder Feeding Rate (g/min)
PC	150	/	10	300	50	8.2
LC	/	1200	5			
PLHC	120	1500	12			

**Table 2:** Elemental distribution of the point scan in Figure 10(c3).

Point (wt%)	C	O	Si	Cr	Fe	Ni	W
P1	7.1	33.8	7.5	1.8	2.7	42.5	4.6
P2	8.4	45.2	6.7	1.2	1.8	33.9	2.8

## Data Availability

The original contributions presented in this study are included in the article. Further inquiries can be directed to the corresponding author.

## References

[1] Vencl A., Gligorijević B., Katavić B., Nedić B., Džunić D. (2013). Abrasive Wear Resistance of the Iron- and WC-based Hardfaced Coatings Evaluated with Scratch Test Method. Tribol. Ind..

[2] Vencl A., Bobi I., Bobi B., Jakimovska K., Svoboda P., Kandeva M. (2019). Erosive wear properties of ZA-27 alloy-based nanocomposites: Influence of type, amount, and size of nanoparticle reinforcements. Friction.

[3] Luo K.X., Yang Q., Wu Z.K., Guo Q.T., Lu J., Luo F.H. (2025). Tailoring high WC addition and crack-free Ni-WC coatings by PTAW: Establishing the relationship between the WC type, its retention and the wear resistance of coatings. Tribol. Int..

[4] Zhao S.B., Jia C.P., Yuan Y.X., Wang L.X., Huang Y.M., Yang L.J. (2022). Effects of laser remelting on microstructural characteristics of Ni-WC composite coatings produced by laser hot wire cladding. J. Alloys Compd..

[5] Li W.Y., Yang X.F., Xiao J.P., Hou Q.M. (2021). Effect of WC mass fraction on the microstructure and friction properties of WC/Ni60 laser cladding layer of brake discs. Ceram. Int..

[6] Azizpour M.J., Rad M.T. (2019). The effect of spraying temperature on the corrosion and wear behavior of HVOF thermal sprayed WC-Co coatings. Ceram. Int..

[7] Zhao G.H., Guo Q.C., Li Y.G., Li J.A., Ma L.F., Li H.Y. (2024). Wear behavior of electron beam remelting modified Ni/WC thermal spray coatings. Surf. Coat. Technol..

[8] Wu T., Shi W.Q., Xie L.Y., Gong M.M., Huang J., Xie Y.P., He K.F. (2023). Study on the effect of Ni60 transition coating on microstructure and mechanical properties of Fe/WC composite coating by laser cladding. Opt. Laser Technol..

[9] Yan X.C., Chang C., Deng Z.Y., Lu B.W., Chu Q.K., Chen X.C., Ma W.Y., Liao H.L., Liu M. (2021). Microstructure, interface characteristics and tribological properties of laser cladded NiCrBSi-WC coatings on PH 13-8 Mo steel. Tribol. Int..

[10] Sun C.W., Kong D.J. (2024). Effects of in–situ laser remelting on structural evolution and high–temperature tribological properties of plasma sprayed Cu11.85Al3.2Mn0.1Ti coating. Mater. Today Commun..

[11] Lan Y., Zhang Y., Peng Y.B., Wang A.D., Gao Y., Yang W.F., Fan W.J., Zhang W., Liu Y. (2024). In-situ synthesis of dual-phase nitrides and multiple strengthening mechanisms in FeCoCrNiAl_0.5_ high entropy matrix composite coatings by laser cladding and plasma nitriding. J. Alloys Compd..

[12] Sun J., Wang H.Y., Liu L.M. (2019). The analysis on the formation of porosity during pulsed laser–Induced TIG hybrid welding of 6061 aluminium alloy at high welding speed. Int. J. Precis. Eng. Manuf..

[13] Zhao S.B., Xu S., Huang Y.M., Yang L.J. (2021). Laser hot-wire cladding of Ni/WC composite coatings with a tubular cored wire. J. Mater. Process. Technol..

[14] Archard J.F. (1953). Contact and Rubbing of Flat Surfaces. J. Appl. Phys..

[15] Chen J.B., Lian G.F., Feng M.Y., Zhang W., Chen R.X. (2025). Microstructure evolution and properties of (Nb,M)C (M = Ti,V and Zr) reinforced Ni-WC coatings by laser cladding. J. Alloys Compd..

[16] Wang H.R., Sun Y.F., Qiao Y.Z., Du X.S. (2021). Effect of Ni-coated WC reinforced particles on microstructure and mechanical properties of laser cladding Fe-Co duplex coating. Opt. Laser Technol..

[17] Peng Y.B., Zhang W., Li T.C., Zhang M.Y., Wang L., Song Y., Hu S.H., Hu Y. (2019). Microstructures and mechanical properties of FeCoCrNi high entropy alloy/WC reinforcing particles composite coatings prepared by laser cladding and plasma cladding. Int. J. Refract. Met. Hard Mater..

[18] Li L.C., Wei X. (2023). Study on the effect of laser cladding composite coating and its WC on crack formation mechanism. Laser Technol..

[19] Ma C.Y., Li H.X., Xia F.F., Yan P. (2024). Preparation and characterization of Ni60-WC composites fabricated using laser cladding technique. J. Mater. Eng. Perform..

[20] Hu C.W., Qi C.Q., Zhao K., Cheng L.Y., Yao W., Li C.G. (2024). The microstructure and wear properties of Ni-coated and agglomerated-sintered WC-10Ni/316 composite by laser melting deposition. Mater. Today Commun..

[21] Erfanmanesh M., Abdollah-Pour H., Mohammadian-Semnani H., Shoja-Razavi R. (2018). Kinetics and oxidation behavior of laser clad WC-Co and Ni/WC-Co coatings. Ceram. Int..

[22] Zhou S.F., Xu Y.B., Liao B.Q., Sun Y.J., Dai X.Q., Yang J.X., Li Z.Y. (2018). Effect of laser remelting on microstructure and properties of WC reinforced Fe-based amorphous composite coatings by laser cladding. Opt. Laser Technol..

[23] Lin G., Cai Z., Lu B., Gu L., Wang Y., Yan X., Qiu H., Guo J., Dong Z., Li F. (2025). Enhanced wear resistance of laser cladded WC-Ni composite coatings by picosecond laser surface texturing. Tribol. Int..

[24] Sanhita P., Bhaskaran N.R., André M. (2023). Effect of tungsten and vanadium additions on the dry abrasive wear and solid particle erosion of flame-sprayed AlCoCrFeMo high entropy alloy coatings. Int. J. Refract. Met. Hard Mater..

[25] Meng Y., Yao F., Zhao L., Li J. (2025). Effect of lap rate on the microstructure and properties of TiCN/Ni60 composite coatings deposited by electromagnetic-assisted laser cladding. Mater. Chem. Phys..

[26] Da Q., Kang J., Ma G., Zhou Y., Fu Z., Zhu L., She D., Wang H. (2024). Microstructure, tribocorrosion mechanism and corrosion property via HVOF prepared AlCoCrFeNi/WC composite coating. Surf. Coat. Technol..

[27] Hamid A.-A., Huiqing F., Mhmood I.A., Mohammed A.-B. (2022). The dry sliding wear rate of a Fe-based amorphous coating prepared on mild steel by HVOF thermal spraying. J. Mater. Res. Technol..

[28] Wang Y., Zhao X., Hao E., Bu Z., An Y., Zhou H., Chen J. (2022). High temperature induced “glaze” layer formed in HVOF-sprayed NiCrWMoCuCBFe coating and its wear reduction mechanism. Friction.

[29] Li S., Chen L., Zhu L., Zhang X., Ren X. (2024). Comparative research on the microstructure and mechanical properties of traditional and induction heating aided extreme-high-speed laser cladding of Ni60 coatings. Appl. Phys. A.

[30] Zhang X., Wang Q., Deng Y., Chai H., Hu S., Feng Y., Xi Y., Dong L., Lin L., Lin Y. (2024). Effect of microstructure and micromechanics on wear/wear-corrosion mechanism of laser-repaired Ni-WC coating. Eng. Fail. Anal..

[31] He S., Yao C., Shin K.-Y., Park S., Shim D. (2024). Microstructure and wear behaviors of a WC10%-Ni60AA cermet coating synthesized by laser-directed energy deposition. Surf. Coat. Technol..

[32] Liu Q.-S., Liu X.-B., Wang G., Liu Y.-F., Meng Y., Zhang S.-H. (2022). Effect of Cu content on microstructure evolution and tribological behaviors of Ni60 composite coatings on 45# steel by laser cladding. Opt. Laser Technol..

[33] Luo K., Ma H., He J., Lu J., He J., Wu N., Li C., Li Y., Luo F. (2024). Wear performance of Ni-WC composites and heat-damage behaviour of WC particle during vacuum-induction melting process. Wear.

[34] Hu Y.J., Wang Z.X., Pang M. (2022). Effect of WC content on laser cladding Ni-based coating on the surface of stainless steel. Mater. Today Commun..

[35] Marta O., Alaitz Z., Iñaki A.J., Iñigo L., Nagore O., Aitzol L. (2023). High-temperature tribological performance of functionally graded Stellite 6/WC metal matrix composite coatings manufactured by laser-directed energy deposition. Friction.

[36] Liu Z.C., He C., Kong D.J. (2024). Effect of powder feeding speed on microstructure and corrosive–wear performance of laser cladded Ni–60%WC coatings. Opt. Laser Technol..

